# The landscape of coding RNA editing events in pediatric cancer

**DOI:** 10.1186/s12885-021-08956-5

**Published:** 2021-11-17

**Authors:** Ji Wen, Michael Rusch, Samuel W. Brady, Ying Shao, Michael N. Edmonson, Timothy I. Shaw, Brent B. Powers, Liqing Tian, John Easton, Charles G. Mullighan, Tanja Gruber, David Ellison, Jinghui Zhang

**Affiliations:** 1 Department of Pathology, St. Jude Children’s Research Hospital, Memphis, TN 38105 USA; 2 Department of Computational Biology, St. Jude Children’s Research Hospital, Memphis, TN 38105 USA; 3 Department of Pediatrics, Stanford University, Palo Alto, California 94305 USA

**Keywords:** RNA editing, Pediatric cancer, Genomics, Immunotherapy

## Abstract

**Background:**

RNA editing leads to post-transcriptional variation in protein sequences and has important biological implications. We sought to elucidate the landscape of RNA editing events across pediatric cancers.

**Methods:**

Using RNA-Seq data mapped by a pipeline designed to minimize mapping ambiguity, we investigated RNA editing in 711 pediatric cancers from the St. Jude/Washington University Pediatric Cancer Genome Project focusing on coding variants which can potentially increase protein sequence diversity. We combined de novo detection using paired tumor DNA-RNA data with analysis of known RNA editing sites.

**Results:**

We identified 722 unique RNA editing sites in coding regions across pediatric cancers, 70% of which were nonsynonymous recoding variants. Nearly all editing sites represented the canonical A-to-I (*n* = 706) or C-to-U sites (*n* = 14). RNA editing was enriched in brain tumors compared to other cancers, including editing of glutamate receptors and ion channels involved in neurotransmitter signaling. RNA editing profiles of each pediatric cancer subtype resembled those of the corresponding normal tissue profiled by the Genotype-Tissue Expression (GTEx) project.

**Conclusions:**

In this first comprehensive analysis of RNA editing events in pediatric cancer, we found that the RNA editing profile of each cancer subtype is similar to its normal tissue of origin. Tumor-specific RNA editing events were not identified indicating that successful immunotherapeutic targeting of RNA-edited peptides in pediatric cancer should rely on increased antigen presentation on tumor cells compared to normal but not on tumor-specific RNA editing per se.

**Supplementary Information:**

The online version contains supplementary material available at 10.1186/s12885-021-08956-5.

## Background

Post-transcriptional modification of RNA sequences, termed RNA editing, occurs in many species [1]. In humans, two canonical editing types have been identified: adenosine to inosine (A-to-I) editing mediated by the adenosine deaminase acting on RNA (ADAR) enzyme family [2] and cytosine to uracil (C-to-U) editing induced by apolipoprotein B mRNA editing (APOBEC) enzymes [3]. While most editing occurs in Alu repeats [4, 5], RNA editing can also affect protein coding regions [5, 6]. RNA-Seq analysis can identify these editing events through comparison with DNA sequencing, including whole genome (WGS) or whole exome (WES) sequencing, by identifying variants present in RNA but not in DNA [7]. Multiple studies have used RNA-Seq data from The Cancer Genome Atlas (TCGA) to analyze RNA editing events in adult solid tumors and their effects on cancer viability, invasiveness, drug sensitivity, and patient survival [8, 9]. However, RNA editing has not been investigated in pediatric malignancies.

Despite clearly established mechanisms for canonical RNA editing, there is a lack of consensus regarding its prevalence. Some consider RNA editing to be a rare event across the transcriptome [10–13], while others consider it widespread [14, 15] perhaps confounded by mistaken inclusion of technical artifacts [11–13]. For example, one study comparing RNA and DNA sequencing of B cell lines, primary skin fibroblasts and cerebral cortex reported abundant exonic RNA editing, including many noncanonical events [14]. However, reanalysis of the same data showed that RNA editing was less frequent [10, 16], and most of the previously reported [14] editing sites were likely the result of faithful transcription of pseudogenes that share high homology with the canonical genes. There is also disagreement regarding the prevalence of non-canonical (non-A-to-I, non-C-to-U) RNA editing [10, 17–19].

These controversies highlight the importance of accurate RNA-Seq mapping to the human transcriptome [20], which can reduce false-positive RNA editing events and increase the sensitivity for detection of true events. RNA-Seq mapping algorithms were initially designed to ascertain gene expression levels but were not optimized for detecting RNA editing events. For example, when sequencing reads end near splice junctions or true RNA editing events, soft-clipped mappings may be produced, hampering detection of editing events. It is also difficult to ensure accurate mapping to paralogs or expressed pseudogenes. To reduce mapping artifacts in RNA-Seq and thus improve the detection of RNA editing, we have developed an alignment pipeline, StrongArm, which performs competitive mapping with multiple aligners to multiple reference databases to resolve ambiguity by applying knowledge-guided rules. These rules were designed to reduce the error rate and bias from a single aligner, especially near error-prone splice junctions and in paralogous regions. This competitive mapping approach, initially designed for detecting complex gene fusion events in ependymoma [21], has the potential for systematically removing false-positive RNA editing calls caused by a variety of sources of error.

We applied StrongArm along with a post-processing pipeline to identify novel and known single nucleotide variant (SNV) RNA editing events in protein-coding regions that show differences in matched RNA-Seq and DNA sequencing (WGS or WES) from 711 pediatric cancer samples from the St. Jude/Washington University Pediatric Cancer Genome Project (PCGP) [22]. As low-quantity RNA editing events may be difficult to detect de novo*,* we also analyzed known RNA editing sites reported in the RADAR [5] database, a well-curated RNA editing resource, as done by others [8]. In all, we identified 722 RNA editing sites in coding regions across pediatric cancers, including 584 known and 138 novel editing sites. We observed an enrichment of RNA editing in pediatric brain tumors, including in genes involved in neurotransmitter signaling. We compared pediatric cancer RNA editing profiles to normal tissues from the GTEx project and found that the coding RNA editing profile of each pediatric cancer type largely resembles that of its corresponding normal tissue. This suggests that RNA editing is rarely tumor-specific in pediatric cancer but is largely related to the tissue of origin. Together, these results present a comprehensive analysis of RNA editing in pediatric cancer, yielding novel RNA editing events whose biological function should be investigated in future studies.

## Methods

### Sample collection

Details regarding RNA and DNA isolation from PCGP samples have been reported in a series of PCGP-related papers [22–31]. All samples have also been published previously and the raw data of the entire PCGP cohort can be accessed via the St. Jude Cloud Genomics Platform (https://pecan.stjude.cloud/permalink/rnaediting) [32].

### RNA-Seq

PolyA-enriched mRNA-seq of PCGP samples was performed using the Illumina TruSeq V2 RNA library preparation kit, with a starting input of 1μg of total RNA according to manufacturer’s protocol. The number of cycles of library amplification was reduced to 10 cycles to reduce PCR duplicates. The resulting data files were converted to FASTQ files using CASAVA 1.8.2. All reads were 101 bp in length. For discovery of RNA editing events, we initially used 717 pediatric cancer samples’ RNA-Seq from the PCGP, including only samples for which tumor-normal DNA-Seq (WGS or WES) was also available (Supplementary Fig. 1). The novel editing sites thus identified, along with known RNA editing events from RADAR, were then analyzed in 954 PCGP samples with RNA-Seq, whether or not DNA-Seq was available. Finally, these 954 samples were filtered to remove samples for which RNA was isolated outside of St. Jude Children’s Research Hospital, due to batch effects in RNA variant allele fractions (VAFs), and relapsed samples and other samples were also filtered out such that only one diagnosis sample per patient was included in the final 711 samples (Supplementary Fig. 1).

### Whole exome and whole genome sequencing

WES was performed using the Illumina TruSeq Exome Library Prep Kit with 1μg of genomic DNA input using the manufacturer’s protocol. The WGS was performed for paired tumor and normal genomes to > 30-fold coverage as described previously [33]. WES and WGS were performed on the Illumina HiSeq 2000 using a paired sequencing (2 × 100 cycles).

### RNA editing validation

Validation of RNA editing was performed by deep amplicon sequencing on the MiSeq platform. Flanking Primers were designed using Batch Primer3 [34] with local batch automation and parameter modification. Optimal amplicon sizes ranged from approximately 120 bp–200 bp for use in downstream library construction. Total RNA was reverse transcript to cDNA using BioRad iScript cDNA Synthesis Kit. PCR was performed using AmpliTaq Gold 360 master mix (Applied BioSystems), 10 μM of each primer, and 10 ng of genomic DNA and cDNA using the following parameters: 95 °C for 10 min, 95 °C for 30 s, 58 °C for 30 s, 72 °C for 30 s for 35 cycles, 72 °C for 5 min, and storage at 4 °C. All amplicons were quality-checked on a 2% agarose E-gel (Invitrogen). Amplicons were pooled and purified using Agencourt Ampure XP Beads. DNA libraries were created from pooled amplicons using the Nextflex DNA kit (Bioo Scientific), following the manufacturer’s instructions. Libraries were normalized for sequencing on Illumina MiSeq platform by using a 2 × 150 paired-end version 2 sequencing kit.

### StrongArm mapping pipeline

**Fig. 1:**
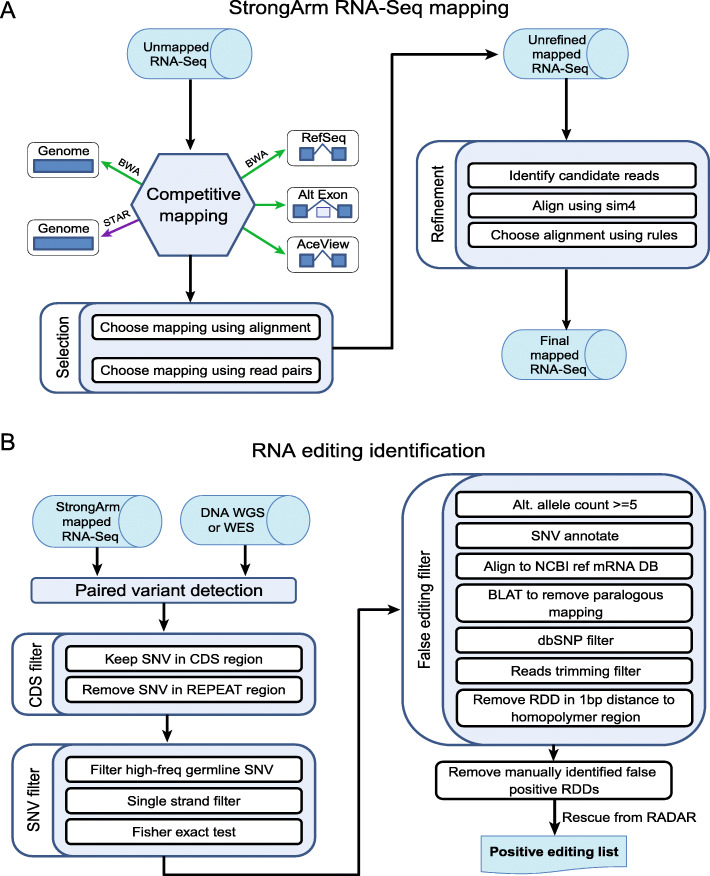
The StrongArm RNA-Seq mapping and RNA editing detection pipelines. **A** Schematic workflow of StrongArm RNA-seq mapping pipeline. The pipeline starts with competitive mapping of 5 different combinations of mapper and database, followed by further local refinement. **B** RNA editing identification pipeline. RNA-Seq BAM files are aligned with StrongArm as shown in (A), and germline and somatic DNA variants are also called from the same patient using WGS or WES of matched tumor and germline DNA. The pipeline searches for RNA-specific (RNA editing) variants in coding (CDS) regions by comparing RNA-Seq reads to DNA-Seq. A series of false editing filters is then employed to remove RNA editing artifacts, followed by manual review of the BAM alignment. The RNA editing candidates are then used to evaluate the editing levels cross the whole cohort

StrongArm accepts reads stored in unaligned BAM format [35], which may be generated from FASTQ using the FastqToSam function of Picard (http://picard.sourceforge.net). We used several input BAMs for each sample, each with two million reads. The whole workflow of StrongArm is illustrated in Fig. 1A. All mapping of PCGP RNA-Seq data was done using the GRCh37 reference genome.

In the first phase, reads were aligned using five combinations of mapper and database. The aligners we used include BWA [36] and STAR [37]. We used the reference genome database and three custom databases. Each sequence in the custom databases is built by selecting a set of exons from a particular annotation source (Table 1). The sequences corresponding to these exons from the forward strand of the reference genome are concatenated to make the custom sequence. Information about the set of exons used is stored in the sequence name. After mapping to the custom databases, each mapping is translated into genomic coordinates using the information from the sequence name, and the reference annotation. The combinations of mapper and database used are listed in Table 1.

**Table 1:** The five mapper and database combinations used by StrongArm

Priority	Mapper	Database
1	BWA	**RefSeq**: Every RefSeq transcript found in UCSC’s refFlat table [38]; useful for finding canonical splicing.
2	BWA	**RefSeq alternate exons**: Fragments of RefSeq transcripts formed by choosing an ordered subset of exons from each gene that contains a single pair of adjacent exons which are not adjacent in any annotation, and with 100 bp of sequence on either side of the event; useful for finding alternate splicing.
3	BWA	**AceView**: Every AceView transcript found in UCSC’s assembly table; useful for various other known splice forms.
4	BWA	**Whole genome**: the genome reference sequence (no translation is performed); useful for unspliced reads and some structural variation events.
5	STAR	**Whole genome (STAR)**: the genome reference sequence (no translation is performed); useful for some novel exons and structural variation events.

After initial mapping, the pipeline chooses a record for each read from among the five available. The best record is chosen according to the following algorithm: (1) mapped records are always better than unmapped; (2) among mapped records, those with more matches are always better than those with fewer; and (3) among mapped records with the same number of matches, records with fewer indels are always better than those with more.

At this stage, there may still be several records tied for the best. If there are multiple candidates, and the reads are paired, then the best pair is chosen based on the following rules: (1) pairs on the same chromosome are always better than pairs on different chromosomes; (2) pairs that are closer (on a log10 scale, with integer granularity) are then preferred; (3) pairs in forward-reverse orientation are then preferred; (4) pairs from the same mapper and database combination are finally preferred; and (5) if ties remain, then the choice is made based on the priority of the mapper and database combination used (Table 1).

Next, the individual aligned BAM files are sorted and merged, duplicates are marked, and the files are indexed using Picard tools SortSam, MergeSamFiles, and MarkDuplicates. The resulting unrefined BAM file is usable, and may be sufficient for some analyses. This completes the alignment phase of the mapping.

The second phase of the mapping is for refinement. First, records of interest are extracted using SAMExtractUnmapped in Bambino [39]. The records extracted include unmapped reads and reads with soft-clipping, indels, or high quality mismatches. The resulting files are converted to FASTA, large files are split, and small files are batched as an optimization.

These reads are then aligned to the reference genome using sim4 [40]. If the mate read is aligned, then the sim4 search is restricted to the 100 kb region on the side of the mate read that would be expected to achieve forward-reverse orientation. If the mate read is not aligned, then the sim4 search is restricted to 100 kb on either side of the original mapping position.

If the sim4 mapping is better than the original, then it is used in place of the original. The algorithm determines if the sim4 alignment is an improvement as follows: (1) if the read was originally unmapped, then it is an improvement; (2) the alignments are scored using + 1 for alignment, − 1 for gap open, − 1 for gap extend, and + 5 for any splice of length < 100 kb, and if the scores are unequal, then improvement is determined based on the scoring; (3) if the reads were previously on different chromosomes, but sim4 places them on the same chromosome, then it is an improvement; (4) if the reads were previously not in forward-reverse orientation, but sim4 places them in forward-reverse, then it is an improvement; and (5) otherwise, it is not an improvement.

At this point, the pipeline also soft-clips the alignment of poly-A tails, which may contain one or more spurious splices to poly-A runs in the reference genome. The new individual aligned BAM files are sorted and merged, duplicates are marked, and the files are indexed, as for the unrefined BAM, to create the final BAM file.

### TopHat and STAR

StrongArm alignment was compared to TopHat and STAR alignment for 15 PCGP samples’ RNA-seq data. TopHat version 2.1.1 using Bowtie version 2.1.0 was run in both default mode with parameter “-m 2″, and annotation-based mode with parameter “-m 2 --transcriptome-index GENCODE.v19.index”. STAR mapping was performed with STAR version 2.7.1a in two-pass mode. The GRCh37 reference genome was used. The RNA editing VAF of the 722 sites analyzed in the study were determined using the Ace2.ReadReport utility within Bambino [39] for the samples aligned with StrongArm, TopHat, and STAR.

### Post-processing for detecting RNA editing events

After mapping is performed with StrongArm, the RNA editing detection pipeline starts with variant calling using Bambino [39] followed by filters to limit analysis to SNVs within gene coding regions and to remove false editing events due to germline variants, paralogous mapping, and homopolymer regions. The resultant RNA editing candidates were further curated by manual review. To rescue low-confidence editing events for which editing was not detected de novo*,* we reviewed the evidence of coding editing sites that were included in the RADAR database with ≥3 mutant reads in ≥2 PCGP samples (a total of 384 RNA editing events were rescued). The RADAR [5] version 2 database (hg19) was used to compare our identified editing events with RADAR. We also determined whether RNA editing events we discovered de novo were already present in DARNED or REDIportal, in addition to RADAR. For this, DARNED [6] hg19 editing sites were downloaded in February 2021 from https://darned.ucc.ie/download/. REDIportal [41] hg19 editing sites were downloaded February 2021 from http://srv00.recas.ba.infn.it/atlas/download.html. The RNA editing VAF of the 722 sites were calculated using the Ace2.ReadReport utility within Bambino [39].

### Normal sample analysis using GTEx

GTEx RNA-Seq files were previously downloaded from dbGaP accession phs000424 and reads were previously mapped using STAR version 2.7.1a in two-pass mode to hg38 for another study. To avoid remapping these 5454 BAM files to hg19 using StrongArm for this study (due to the storage resources and computational throughput this would require) we instead used the existing hg38-aligned files and analyzed the RNA editing levels for each of 722 RNA editing sites identified in pediatric cancer (converted to hg38 coordinates), using the Ace2.ReadReport utility in Bambino [39]. To verify that RNA editing results would have been similar had we re-mapped all GTEx samples to StrongArm with hg19, we correlated RNA editing in 18 GTEx samples re-mapped with StrongArm (hg19) vs. the existing STAR (hg38) alignments. In each of these 18 samples, the Pearson r correlations for total read coverage across the 722 sites when comparing the two approaches were r > 0.97 and the mutant read correlations were r > 0.99, indicating similar results between the two approaches. Sample tissue type annotations were obtained from https://github.com/ucscGenomeBrowser/kent/blob/master/src/hg/makeDb/outside/gtexHub/metadata/sraToSample.tab.

### High-confidence RNA editing events

**Fig. 2:**
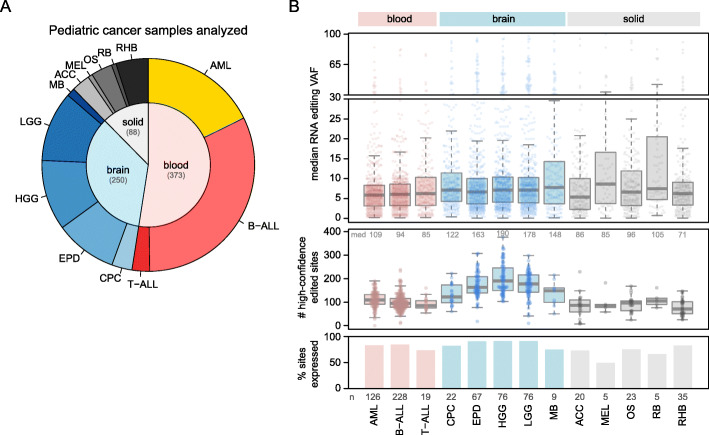
Analysis of RNA editing in pediatric cancers. **A** Pie chart showing the number and type of pediatric cancer samples analyzed, including 711 samples from the PCGP (excluding those showing batch-specific effects in RNA editing VAFs). Samples are divided into blood, brain, and solid (extracranial) cancers and include only diagnosis samples, with one sample per patient. Subtypes of blood, brain, and solid tumors include acute myeloid leukemia (AML), B- and T-acute lymphoblastic leukemia (B-ALL and T-ALL), choroid plexus carcinoma (CPC), ependymoma (EPD), high-grade and low-grade glioma (HGG and LGG), medulloblastoma (MB), adrenocortical carcinoma (ACC), melanoma (MEL), osteosarcoma (OS), retinoblastoma (RB), and rhabdomyosarcoma (RHB). **B** RNA editing VAFs for each cancer type. The 722 RNA editing sites mentioned in the text were analyzed. Bottom panel y-axis indicates the percentage of the 722 variants in each cancer type that were expressed in at least 3 samples (with 10 reads of coverage), and the numbers at bottom indicate the total number of samples analyzed in each cancer type. The middle panel shows the number of RNA editing sites in each sample (point) that were edited with high confidence (Methods). Median values are shown at the top of the plot. Boxplot shows median (thick center line) and interquartile range (box). Whiskers are described in R boxplot documentation (a 1.5*interquartile range rule is used). In the top panel, the y-axis represents the median RNA VAF, such that each point represents the median RNA VAF for one specific RNA editing site in one specific cancer type. Only positively edited samples were included in the quantification of the median VAF (zero-VAF samples excluded) and only high-confidence editing events were included (Methods). Boxplots median, interquartile range, and whiskers are as in middle panel. Only RNA editing sites for which at least 3 samples in the cancer type had at least 10 reads of RNA-Seq coverage are shown

In Fig. 2B boxplots, only high-confidence RNA editing events were shown, and high confidence was defined as follows. If an editing site had read coverage of greater than 100, at least 3 mutant reads were required to consider the site edited with high confidence. For sites with 20–99 reads of coverage, at least 2 mutant reads were required. Finally, for sites with less than 20 reads of coverage, only 1 mutant read was required to consider the site high confidence. These thresholds were based on analysis of adjacent control sites that were within 2 base pairs upstream or downstream of the 722 RNA editing sites in 15 PCGP samples’ RNA-Seq data, to quantify the background error mutation rate and thus determine what number of mutant reads indicated true-positive editing. Of the 722 RNA editing sites, 569 had a suitable adjacent control site within 2 base pairs upstream or downstream of the editing site that was of the same reference allele (e.g. A for an A > G variant) as the actual editing site. We evaluated the mutation error rate using these 569 adjacent control sites and found that among RNA editing sites with less than 20 reads of coverage, 94% of 1-mutant read variants at RNA editing sites were true-positives. However, below 100 reads of coverage, only 79% of 1-mutant read variants at RNA editing sites were true-positives, and thus 2 mutant reads were required when 20–100 reads of coverage were available (leading to 90% true-positives). Above 100 reads of coverage, sites with 3 mutant reads gave over 89% true positives, whereas 2 mutant reads gave only 76% true positives, leading to 3 mutant reads required above 100 reads of coverage.

### Neoepitope prediction

Neoepitope prediction was performed using neoepiscope [42] run on all non-silent RNA editing events identified, using the chromosome, position, reference allele, and alternate allele of each editing event as input. Predictions were made for 15 common HLA haplotypes reported in the literature [43, 44].

## Results

### Competitive RNA-Seq mapping using StrongArm

Current RNA-Seq alignment tools have varying properties that can affect the detection of RNA editing. For example, STAR has a high mapping rate because it permits incomplete alignments which increase the sensitivity for detecting RNA editing events, but the fraction of fully-mapped reads is lower than other tools which can lead to false-negatives. Annotation-based TopHat2 has the opposite characteristics [45]. As expression of pseudogenes and paralogs is common across the human genome, ambiguity in RNA-Seq mapping can lead to erroneous RNA editing calls [10, 16], whereas mapping of DNA sequencing reads can rely on unique intronic sequences to produce the correct mapping. Moreover, aligners like STAR will occasionally soft-clip a read when the read’s end spans a splice junction (Supplementary Fig. 2A) or contains bona fide nucleotide variants (e.g. RNA editing or genetic variants). For RNA editing analysis, this can lead to missing true RNA editing events (Supplementary Fig. 2B).

To facilitate the detection of RNA editing and other variants using RNA-Seq data, we designed an exhaustive mapping pipeline called StrongArm. This pipeline selects the mapping location of a read-pair based on mapping to five different reference database/mapper combinations, from which the best mapping is chosen (Fig. 1A). To analyze the pipeline’s sensitivity, we compared StrongArm results with two popular aligners, STAR and TopHat2, using RNA-Seq data from 15 pediatric cancer samples. StrongArm and STAR had higher mapping rates than TopHat2 or annotation-based TopHat2 (Supplementary Fig. 3A), with the algorithmic trade-off of more soft-clipped reads with StrongArm and STAR (Supplementary Fig. 3B). Compared to STAR, StrongArm was able to map more reads at full length without soft-clipping (Supplementary Fig. 3B, C), largely due to improved mapping around splice junctions and non-reference genomic locations (Supplementary Fig. 2). StrongArm alignment also led to significantly more RNA editing sites being evaluable (which we defined by at least 10 reads of coverage at the editing site, as done by others [8]) than STAR (Supplementary Fig. 4A), although when coverage was evaluable with both tools the RNA editing VAFs were comparable (Supplementary Fig. 4B).

### Identification of coding RNA editing events in pediatric cancers

We analyzed RNA editing in 954 pediatric cancer samples from PCGP (later filtered to 711 as described in Methods; Fig. 2A, Supplementary Fig. 1) using RNA-Seq data mapped with StrongArm (Fig. 1A). We first identified RNA-specific single-nucleotide variants (SNVs) from the aligned RNA-seq reads using Bambino [39], followed by applying multiple filters to remove false-positives (Fig. 1B, Methods). The pipeline eliminates germline and somatic DNA variants from consideration using matched tumor-normal WGS or WES (available for 717 cases) and public single nucleotide polymorphism (SNP) data (Fig. 1B, Methods). We focused on variants in coding regions for this analysis.

To remove additional false positive hits, we performed manual curation by visually inspecting the alignment of each variant [39] (Fig. 1B, final step) to remove the remaining false positives in the following categories. (1) Co-occurrence of SNPs with paralogous variants. For example, a false-positive RNA editing call in the mono-exonic *GLUD2* gene was in fact a SNP (rs9421572) in its multi-exonic paralog *GLUD1* near an exon-intron boundary (described in Supplementary Fig. 5A). This error was due to the gap-open penalty incurred by a splice junction in *GLUD1* resulting in a preference for an unspliced region in *GLUD2* during mapping. (2) Variants at the 3′ side of homopolymers on the antisense strand, suggesting an error introduced during reverse transcription (Supplementary Fig. 5B). (3) Imperfect genome annotation. For example, some purported RNA editing events could be accounted for by alternative splicing to exons not included in the transcript model during mapping, thus leading to spurious variant calls, as in the case of *PHB2* (Supplementary Fig. 5C). Manual curation is an important step as automated analysis identified 334 to 1530 (731 on average, after removing somatic SNVs) putative RNA editing events per sample based on de novo variant calling. However, 95 to 99% (99% on average) of these were recognized to be artifacts during manual review. A total of 340 unique editing sites were detected in the PCGP cohort.

Combining these editing sites detected de novo with those present in the RADAR database (Methods), we identified 722 unique RNA editing sites in coding regions post manual curation (Supplementary Table 1), including 706 canonical A-to-I events and 14 canonical C-to-U events. Approximately 70% of these were non-synonymous missense (498), nonsense (1), or stop-loss (8) variants while the remaining 30% were synonymous. Further, 584 of 722 (81%) were previously reported in RNA editing databases (e.g. RADAR [5], DARNED [6], and/or REDIportal [41]). Of the remaining 138 sites not found in these databases, 90 represented a new variant affecting a gene known to have editing events in these databases (e.g. missense but at a different amino acid), while 48 sites represent the first reported RNA editing event affecting protein-coding (e.g. missense, nonsense, or stop-loss) of the gene. We selected 10 editing sites and performed experimental validation by deep amplicon sequencing using paired DNA/RNA samples in five leukemias with varying RNA VAFs; all variants were confirmed to be present exclusively in RNA samples (Supplementary Table 2).

We then analyzed the prevalence of RNA editing on these 722 sites across the major subtypes of pediatric cancer, including blood, brain, and solid (extracranial) cancers (Fig. 2A). Brain cancers had more editing events than other cancers (Fig. 2B, middle panel), with a median of 190 positive RNA editing events per sample in high-grade glioma (HGG) and 178 in low-grade glioma (LGG), as compared to blood cancers (from median 85 in T-ALL to 109 in AML) and solid tumors (from median 71 in rhabdomyosarcoma (RHB) to 105 in retinoblastoma (RB)). RNA editing VAFs were low overall in edited transcripts, with similar VAFs across cancer types (Fig. 2B, top, which shows median VAFs at approximately 0.05 for each RNA editing site in each cancer).

### Comparison of RNA editing between pediatric cancers and normal tissue

**Fig. 3:**
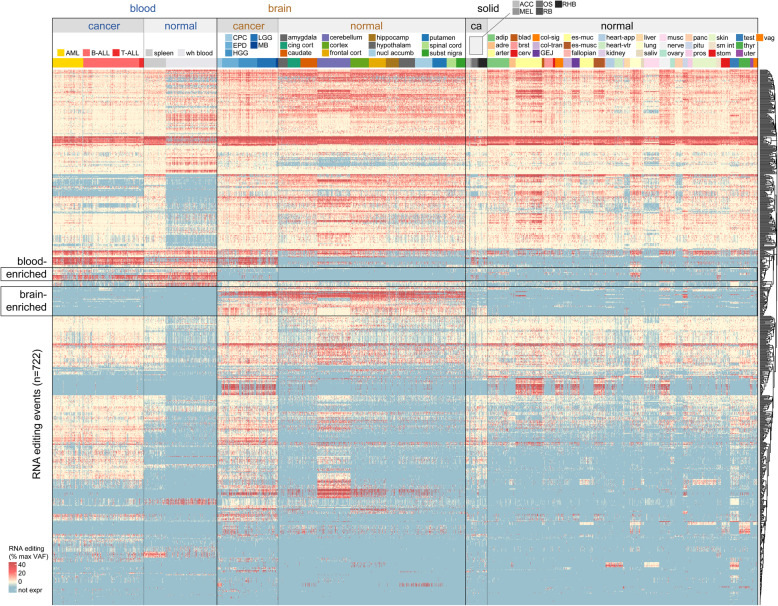
Landscape of RNA editing events in pediatric cancer compared to normal samples. Heatmap showing the level of RNA editing for the 722 editing events reported in the text. Each row represents one of the 722 RNA editing sites. Each column represents one cancer (PCGP, *n* = 711) or normal (GTEx, *n* = 2164 of the 5454 total samples analyzed are shown, as the solid normal tissue samples were thinned to approximately one-fourth the original number for easier viewing) RNA-Seq sample. In the heatmap, red color indicates higher editing level (as a percentage of the max RNA VAF for that variant/row), cream color indicates low editing level, and blue color indicates the expression was too low to evaluate (fewer than 10 reads of coverage at the genomic locus). Samples from the same cancer or normal tissue type are grouped together and color-coded as indicated in legends at top. The name of each cancer and normal tissue type is abbreviated, and the key for determining the full name of each cancer and normal tissue type is in Supplementary Table 6

We next asked whether the 722 RNA editing sites identified in pediatric cancer were also found in normal tissues by analyzing 5454 RNA-Seq samples from GTEx. Only 7 of the 722 RNA editing events were tumor-specific, as the remainder could be found in one or more normal tissues (Fig. 3, Supplementary Table 3); 6 of these 7 sites were only edited in a few cancer samples, while the 7th (a *MEX3C* A74A silent variant) was edited specifically in blood cancers (Supplementary Tables 3–4). We observed RNA editing events which were enriched in both normal and leukemic blood samples (Fig. 3, top box), and events enriched in both normal and cancerous brain samples (Fig. 3, bottom box). This suggests that RNA editing in pediatric cancer is related to the tissue of origin, rather than tumor-specific effects. Some tissue specificity of RNA editing was related to tissue-specific expression of the edited gene, rather than enrichment of editing per se (blue in Fig. 3 indicates lack of expression; see also specific variants discussed below).

**Fig. 4:**
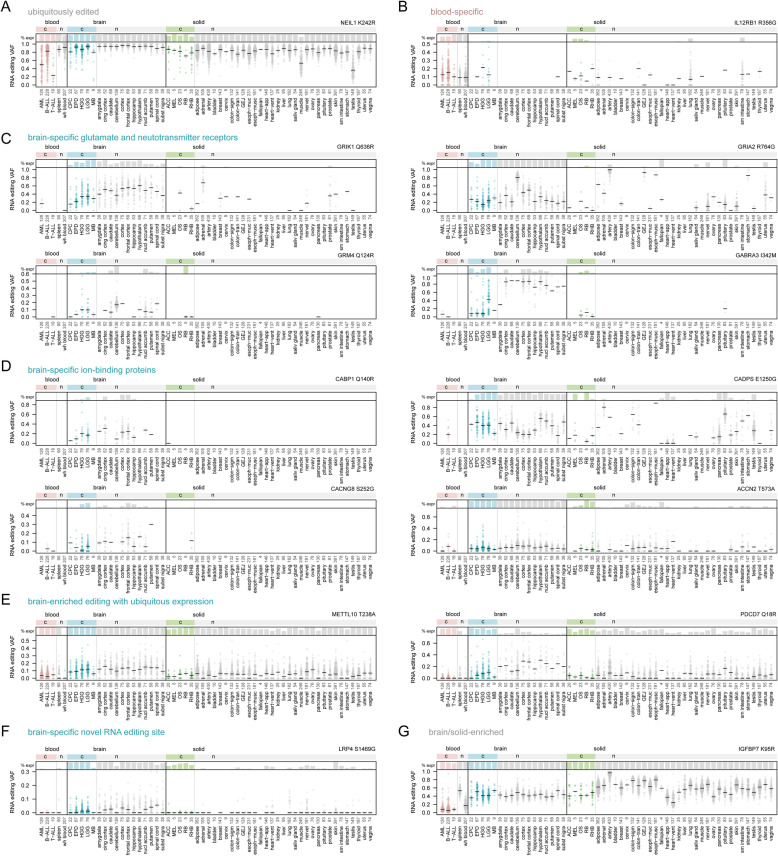
Tissue specificity of selected RNA editing events. Plots are shown for selected RNA editing events taken from among the 722 sites identified in the study. Bottom y-axis for each graph represents the RNA editing VAF for the variant noted. Each point represents one cancer or normal sample, and horizontal black bars represent the median for each cancer or normal tissue type. The top portion of each graph indicates the percentage of samples in which the editing site is expressed (“% expr”) with at least 10 reads of sequencing coverage. VAFs are only shown in the bottom panels for samples meeting this criterion. Samples are divided into cancer (c) or normal (n) tissue types as in Fig. 3 (see Supplementary Table 6 for cancer type abbreviation definitions). The RNA editing site is shown at the top-right of each graph, expressed as the gene and amino acid change caused by the editing event. Each panel highlights an editing event with a specific pattern of interest, including (**A**) a ubiquitously edited site, (**B**) a site both edited and expressed primarily in blood cells, (**C** and **D**) sites both expressed and edited preferentially in the brain, (**E**) sites with ubiquitous expression and editing enrichment in brain, (**F**) a brain-enriched editing site not reported in RADAR, DARNED, or REDIportal, unlike the others in this figure, and (**G**) a site edited preferentially in solid and brain tissue but not edited in most blood tissues

Figure 4 shows the tissue-specific profiles of example RNA editing events with patterns of interest, including ubiquitous editing with ubiquitous expression; tissue-specific editing with tissue-specific expression; and tissue-specific editing with ubiquitous expression. For example, some RNA editing events were ubiquitous across tissue types in genes with ubiquitous expression, such as previously reported *NEIL1* K242R [46] editing (Fig. 4A). Some were enriched in normal blood and leukemic samples due to tissue-specific expression of the gene, including *IL12RB1* R356G (Fig. 4B), detected previously in normal immune cells [47]. Glutamate receptors, including *GRIK1*, *GRM4*, and *GRIA2*, and other receptors involved in neurotransmission were also enriched in both normal and malignant brain samples (Fig. 4C), consistent with studies in normal neural tissue [48]. RNA editing in various calcium and other ion-binding proteins, which can also affect neurotransmission, were likewise enriched in brain samples (Fig. 4D) as expected [5, 6, 49]. The above tissue-specific effects were related to increased expression of the edited gene itself, not necessarily increased RNA editing. By contrast, a few genes including *METTL10* and *PDCD7* had widespread expression across most tissue types, but brain-enriched editing (Fig. 4E), suggesting an increase in editing itself in brain tissue. While the above editing sites are previously reported, a few novel sites showed tissue-specific editing, such as the brain (glioma)-enriched *LRP4* gene (Fig. 4F). Finally, some RNA editing sites showed depletion in a specific tissue type, such as the lack of *IGFBP7* editing in normal and leukemic blood samples (Fig. 4G).

Previously reported editing events in *GRIA2*, *AZIN1*, and *COG3* are thought to promote adult tumor progression based on functional cell viability assays [8]. We also detected these events in multiple pediatric cancer types, suggesting they may promote pediatric cancer progression as well, although they are also present in multiple normal tissues (Supplementary Tables 3–4).

### Association between RNA editing and splicing

RNA splicing and editing can occur co-transcriptionally and RNA editing may alter splicing [50, 51]. This could lead to different editing levels between mRNA and pre-mRNA, which can be analyzed by comparing editing levels among spliced mRNA and unspliced (intron-retaining) pre-mRNA. While we used polyA-enriched RNA-Seq data to analyze PCGP samples, we nonetheless observed substantial intronic reads in many genes, indicating the presence of some pre-mRNA with which this analysis could be performed.

**Fig. 5:**
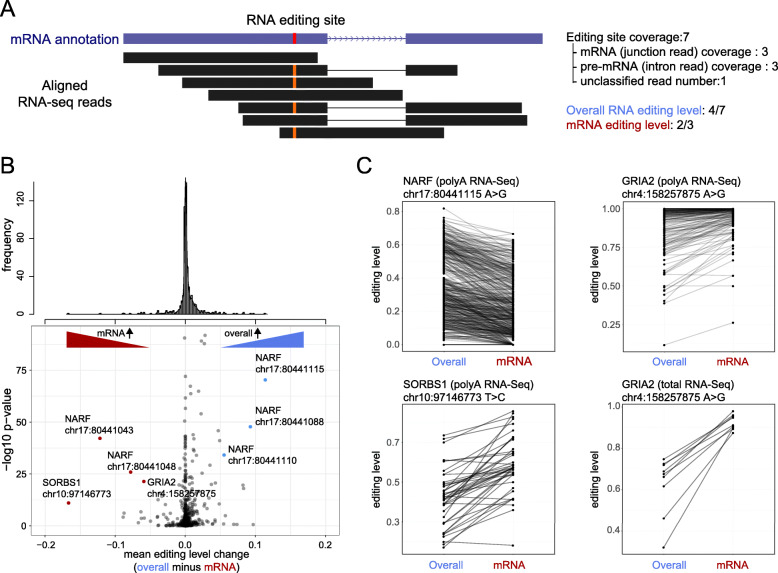
RNA editing differs between total RNA compared to mRNA. (**A**) A method to calculate junction-corrected RNA editing level. To strictly calculate the RNA editing levels using mRNA reads, we only kept reads for which the read or its mated pair were mapped to splice junctions. The “spliced reads” were then used to calculate the mRNA editing levels. (**B**) Difference between overall RNA editing level and junction corrected editing level. Top, the histogram of mean editing level change. Bottom, volcano plot illustrating the difference and *p*-values for each editing site, comparing overall to (junction-corrected) mRNA editing levels. (**C**) Examples of editing sites showing significant editing level change comparing overall RNA editing level to mRNA editing level. The overall and mRNA editing level from the same sample are connected with black lines to indicate paired connections

To assess the spliced mRNA editing level (rather than the overall RNA editing level used in our previous analyses), we first identified the reads for which the read or its mate pair were mapped to splice junctions. These “spliced reads” were considered as a signal of mRNA which were then used to calculate the (spliced) mRNA editing level (Fig. 5A). The difference between the overall editing level (VAF) and mRNA editing level (VAF) were compared using a Wilcoxon Rank Sum test for each editing site.

As shown in Fig. 5B, since polyA-enriched RNA-Seq filters out many immature RNAs, most RNA editing sites do not show significant differences between the overall RNA editing level and the spliced mRNA (junction-corrected) editing level. However, several editing sites showed a significant difference. For example, *NARF* (Fig. 5C, top-left) had a lower editing level in mRNA, but higher overall editing level, suggesting a relationship between editing and splicing. This could be because unedited *NARF* pre-mRNAs are more prone to splicing, or because edited *NARF* mRNAs are more prone to degradation. By contrast, *SORBS1* (Fig. 5C, bottom-left) has more editing in mRNA, possibly because edited *SORBS1* pre-mRNA is more prone to splicing.

Because the junction-corrected mRNA level is calculated from polyA-enriched RNA-Seq in these cases, the real difference between mRNA and pre-mRNA might be more apparent if total RNA library preparation was used. Therefore, we compared a subset of samples sequenced by both total stranded and polyA-enriched RNA-Seq. The *GRIA2* gene, for example, had moderately (though significantly) different editing between mRNA and pre-mRNA using polyA-enriched RNA-Seq (Fig. 5B; Fig. 5C, top-right). However, in total stranded RNA-Seq the different was much more apparent (Fig. 5C, bottom-right), indicating that more splicing-editing correlations may exist than our polyA-focused analysis suggests. Because RNA editing relies on local RNA secondary structures, it is possible that editing preferentially targets spliced transcripts in some genes, and unspliced in others, based on secondary structure. Moreover, editing can create cryptic GT-AG splicing sites to affect splicing output and efficacy. Thus, the interplay between splicing and RNA editing is likely to be gene-specific, consistent with our results (Fig. 5C).

## Discussion

We used a competitive mapping approach to improve paralog mapping and mitigate alignment errors when reads span splice junctions for discovery of RNA editing. This study is the first comprehensive analysis of RNA editing in pediatric cancers, which has been previously studied in adult cancers [8, 9]. We have noted both known and novel RNA editing sites whose biological function could be investigated in future studies.

It has been proposed that RNA editing may lead to neoepitopes that may be targeted by anti-cancer immunotherapies [52]. Indeed, we used neoepiscope [42] to run neoepitope prediction on peptides resulting from RNA editing events to identify peptides predicted to be presented effectively by 15 common HLA class I haplotypes [43, 44]; many of these peptides were predicted to bind effectively to these HLA alleles and thus would theoretically generate epitopes that could be recognized by T cells (Supplementary Table 5). However, our results indicate that, in pediatric cancer, RNA editing events are not specifically enriched in tumors, which would make immunotherapeutic targeting difficult. Rather, nearly all RNA editing events we detected in pediatric cancer were also present in one or more normal tissues; indeed, the profile of each pediatric cancer type essentially matched that of its normal tissue of origin. This suggests that immunotherapeutic efforts focused on identifying tumor-specific RNA editing will be difficult to implement in pediatric cancer. However, if RNA-edited genes are overexpressed in a specific cancer type compared to normal, it may be possible to target these editing events immunotherapeutically with good therapeutic index. For example, the *CD6* gene is overexpressed in a subset of leukemias, with expression of over 100 transcripts per million (TPM) in 10% of acute myeloid leukemia (AML), 18% of T-lineage acute lymphoblastic leukemia (T-ALL), and 2% of B-ALL, but virtually no expression in other cancerous tissues (Supplementary Fig. 6A). *CD6* undergoes RNA editing leading to an S52G missense variant (Supplementary Tables 1, 3, 4), and thus targeting this peptide immunotherapeutically may have therapeutic potential. *CD6* is also overexpressed in some normal blood and small intestinal tissues from GTEx (Supplementary Fig. 6B) concomitant with S52G editing (Supplementary Tables 3, 4), but this may be acceptable given that transient immunosuppression or gastrointestinal issues may be an acceptable toxicity of anticancer therapy.

Further, if certain RNA editing events are preferentially presented via HLA molecules on tumor cells in preference to normal cells, these may represent valid targets (despite RNA editing in both normal and tumor cells) as shown for *CCNI* editing in some adult cancers [53], an editing event which we also observed in all pediatric cancer types analyzed (Supplementary Tables 3–4). While beyond the scope of this study, it would be of interest for future studies to test whether any of the RNA editing events we identified are preferentially HLA-loaded on tumor cells compared to normal cells to identify potential therapeutic targets.

## Conclusions

These findings indicate that the RNA editing profile of each pediatric cancer type is similar to its corresponding normal tissue of origin. Thus, the somatic mutations present in pediatric cancers do not appear to promote tumor-specific RNA editing events which can be immuno-therapeutically targeted. Rather, RNA editing profiles in pediatric cancer likely result from the transcriptional state the cancer inherits from the original normal tissue. However, RNA editing events occurring in cancers with overexpression of the edited transcript may provide therapeutic targets, which merits further study. These results also provide a map of the coding RNA editing events across pediatric cancers, and identify novel RNA editing events whose function should be explored in future studies.

## Supplementary Information

**Additional file 1: Supplementary Fig. 1.** RNA editing analysis workflow and samples included. Workflow showing the discovery process and the criteria for sample inclusion at each step. To discover de novo potentially novel RNA editing sites (top), SNV calling was performed on samples with tumor RNA-Seq, and germline plus tumor DNA-Seq (either WGS or WES), including 717 samples (including 716 pediatric cancer samples from the PCGP and one leukemia cell line, Nalm6). After filtering and discovering RNA editing events in these 717 samples as shown in Fig. 1B, the presence of each RNA editing event discovered de novo or rescued from RADAR (*n* = 722 sites total) was analyzed in 954 pediatric cancer RNA-Seq samples from the PCGP, whether or not DNA-Seq was available (middle). Finally, certain samples with batch effects in RNA editing VAFs and relapsed or duplicate samples were filtered out, resulting in 711 pediatric cancer diagnosis samples with one diagnosis sample per patient (bottom). This set of 711 samples was the primary sample set shown in analyses in this study. **Supplementary Fig. 2.** Examples comparing alignment between StrongArm vs. STAR mapping. (**A**) Splice junction-adjacent reads that failed mapping by STAR (blue) but mapped by StrongArm (red) as viewed in the BAM alignment. This shows a view of one sample’s BAM files aligned by the two tools, with each row representing one read, and part of the *COPA* gene is shown. Soft-clipped read regions have a darker gray appearance. Gray arrows (>) indicate that part of the read is aligned elsewhere (to another exon). Non-reference sites are shown in red, including an RNA editing site (T > C). Only reads containing the edited allele are shown. (**B**) Read with RNA editing at the end that failed mapping (soft-clipped) by STAR but mapped by StrongArm. Coloring and other features are as in (A), except that the *MRPS27* gene is shown. This shows a view of one sample’s BAM files aligned by the two tools. Only reads containing the edited allele (G > A, red) are shown. **Supplementary Fig. 3.** Mapping rate and soft-clipping comparison between StrongArm and other tools. (**A**) Mapping rate comparison between StrongArm, STAR, TopHat2 and annotation-based TopHat2. The mapping rate of the same sample in different aligners is connected by a dotted line. Y-axis represents the percent of mapped reads, and 15 selected pediatric cancer samples from PCGP are analyzed. (**B**) The percentage of reads with soft-clipped nucleotides in each aligner. The same sample is connected by a dotted line. TopHat2 does not map soft-clipped reads. (**C**) The percentage of reads (y-axis) with different numbers of soft-clipped bases (x-axis). The unclipped reads (0) are separated due to different axis scale. This compares STAR (blue points) and StrongArm (red points) and does not include TopHat2, since TopHat2 does not map soft-clipped reads. Each point represents one of the 15 PCGP samples shown in (A) and (B). Boxplot shows median (thick center line) and interquartile range (box). Whiskers are described in R boxplot documentation (a 1.5*interquartile range rule is used). **Supplementary Fig. 4.** Comparison of RNA editing detection between STAR- and StrongArm-mapped RNA-Seq data. This analysis includes 15 PCGP samples’ RNA-Seq data mapped by both StrongArm and STAR (the same samples used for Supplementary Fig. 3). (**A**) More RNA editing sites are evaluable by StrongArm than by STAR mapping. Boxplot shows the number of RNA editing sites (among the 722 RNA editing sites evaluated in the study) that are informative (at least 10 reads of coverage) only by STAR or only by StrongArm mapping in each sample. Boxplot shows median (thick center line) and interquartile range (box). Whiskers are described in R boxplot documentation (a 1.5*interquartile range rule is used). (**B**) High concordance of RNA editing VAFs derived from STAR-mapped and StrongArm-mapped BAM files, when analyzing RNA editing sites with at least 10 reads of coverage with both STAR and StrongArm. See table at top-left of each graph to see the number of variants falling into this category (the “Both (shown)” category). Each graph shows one leukemia patient. Each point represents a single RNA editing event positioned by its RNA editing VAF by StrongArm (x-axis) or by STAR (y-axis). Dotted line represents the identity line (x = y). r value is by Pearson correlation. **Supplementary Fig. 5.** False-positive RNA editing examples requiring manual removal. (**A**) Example false RNA editing due to the presence of a germline SNP in one of two paralogs. *GLUD1* and *GLUD2* are paralogs; *GLUD1* has introns while *GLUD2* is mon-exonic. The coding regions (CDS) have 98% identity between the two genes. Left, in patients with *GLUD1* SNP rs9421572 (a SNP which is near a splice junction in exon 7), DNA (WGS) reads will correctly map the SNP to *GLUD1* instead of *GLUD2*, as the intronic region of reads containing the SNP will map uniquely to *GLUD1* but not to intron-less *GLUD2*. Right, in RNA-Seq, by contrast, neither *GLUD1* nor *GLUD2* mRNAs contain introns, and therefore the mapping will prefer *GLUD2* since mapping that does not require the read to span a splice junction is preferred, and *GLUD2* lacks introns making it preferred. The *GLUD1* SNP is therefore aberrantly considered to be a *GLUD2* RNA editing event. Such events must be removed by manual curation. (**B**) Example of false RNA editing due to the presence of a homopolymer genomic region using an example in the *RNF19A* gene. False-positive editing events frequently occurred within one base position of homopolymer sequences on the 3′ side of the homopolymer along the antisense strand, suggesting the error was introduced during reverse transcription. Such events must be removed by manual curation. (**C**) Example of false RNA editing due to mapping to the wrong splice variant. The *PHB2* gene includes a very small (or “nano”) exon in one of the UCSC transcripts (uc021qug.1). However, none of the RefSeq transcripts used for mapping includes this exon; therefore, reads which include the nano exon are instead incorrectly mapped to NM_001144831 which lacks the nano exon. Thus, RNA-Seq reads for many samples, including the example adrenocortical cancer sample SJACT001, incorrectly map to NM_001144831 and appear to have an RNA editing event in a region that should in fact map to the nano exon. **Supplementary Fig. 6.** An example of outlier expression of an RNA-edited gene in a specific tissue type. (**A**) Boxplot showing *CD6* expression in TPM across PCGP pediatric cancer tissues. Parentheses indicate the number of samples analyzed. See Fig. 2 legend for cancer type abbreviations. Each blue point represents one cancer sample. Outlier samples, as determined by the R “boxplot” function, are also outlined in red. Boxplot shows median (thick center line) and interquartile range (box). Whiskers are described in R boxplot documentation (a 1.5*interquartile range rule is used). (**B**) As in (A), except that the expression of *CD6* is shown in normal GTEx tissues. Due to different RNA-Seq library preparation and sequencing methods, the data in panels (A) and (B) may not be directly comparable to one another.

**Additional file 2: Supplementary Table 1.** 722 RNA editing sites identified in pediatric cancer samples. **Supplementary Table 2.** Deep amplicon sequencing of RNA vaildation of 10 RNA editing events. **Supplementary Table 3.** Percentages of cancer and normal tissue samples with RNA editing at each site. **Supplementary Table 4.** VAFs of RNA editing events in each sample. **Supplementary Table 5.** Neoepitope predictions for 722 RNA editing sites. **Supplementary Table 6.** Abbreviations for cancer and normal tissue types.

## Data Availability

Raw sequencing data for all cancer samples are available on St. Jude Cloud [32] (https://pecan.stjude.cloud/permalink/rnaediting) and BAM files are available on EGA at accessions referenced in past PCGP-related studies [22–31] (EGAD00001000261, EGAD00001001864, EGAD00001000260, EGAD00001000161, EGAD00001000259, EGAD00001000164, EGAD00001000160, EGAD00001000163, EGAD00001000268, EGAD00001000162, EGAD00001000085, EGAD00001000165, EGAD00001000135, EGAD00001000159). GTEx data are available on dbGaP under accession phs000424.v8.p2.

## Abbreviations

ACC: Adrenocortical carcinoma
ADAR: Adenosine deaminase acting on RNA
AML: Acute myeloid leukemia
APOBEC: Apolipoprotein B mRNA editing enzymes
B-ALL: B-lineage acute lymphoblastic leukemia
CDS: Coding regions
CPC: Choroid plexus carcinoma
EPD: Ependymoma
GTEx: Genotype-Tissue Expression project
HGG: High-grade glioma
LGG: Low-grade glioma
MB: Medulloblastoma
MEL: Melanoma
OS: Osteosarcoma
PCGP: St. Jude/Washington University Pediatric Cancer Genome Project
RB: Retinoblastoma
RHB: Rhabdomyosarcoma
SNP: Single nucleotide polymorphism
SNV: Single nucleotide variant
T-ALL: T-lineage acute lymphoblastic leukemia
TCGA: The Cancer Genome Atlas
TPM: Transcripts per million
VAF: Variant allele fraction
WES: Whole-exome sequencing
WGS: Whole-genome sequencing
