# Supplementary material for: The landscape of coding RNA editing events in pediatric cancer
Source: BMC Cancer. 2021 Nov 17;21:1233. doi: 10.1186/s12885-021-08956-5 (PMC8597231; doi:10.1186/s12885-021-08956-5)

Supplementary Figure 1

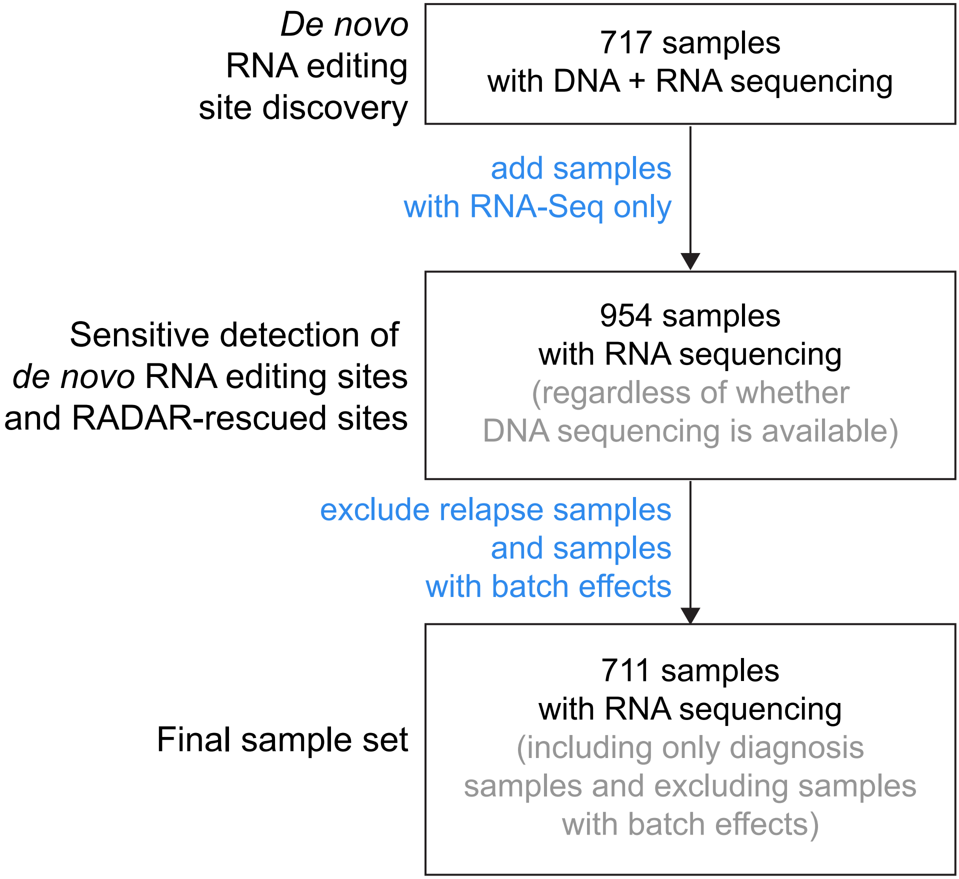

Supplementary Figure 2

A

Junction reads that failed mapping by STAR but mapped by StrongArm
(only reads containing edited allele are shown)

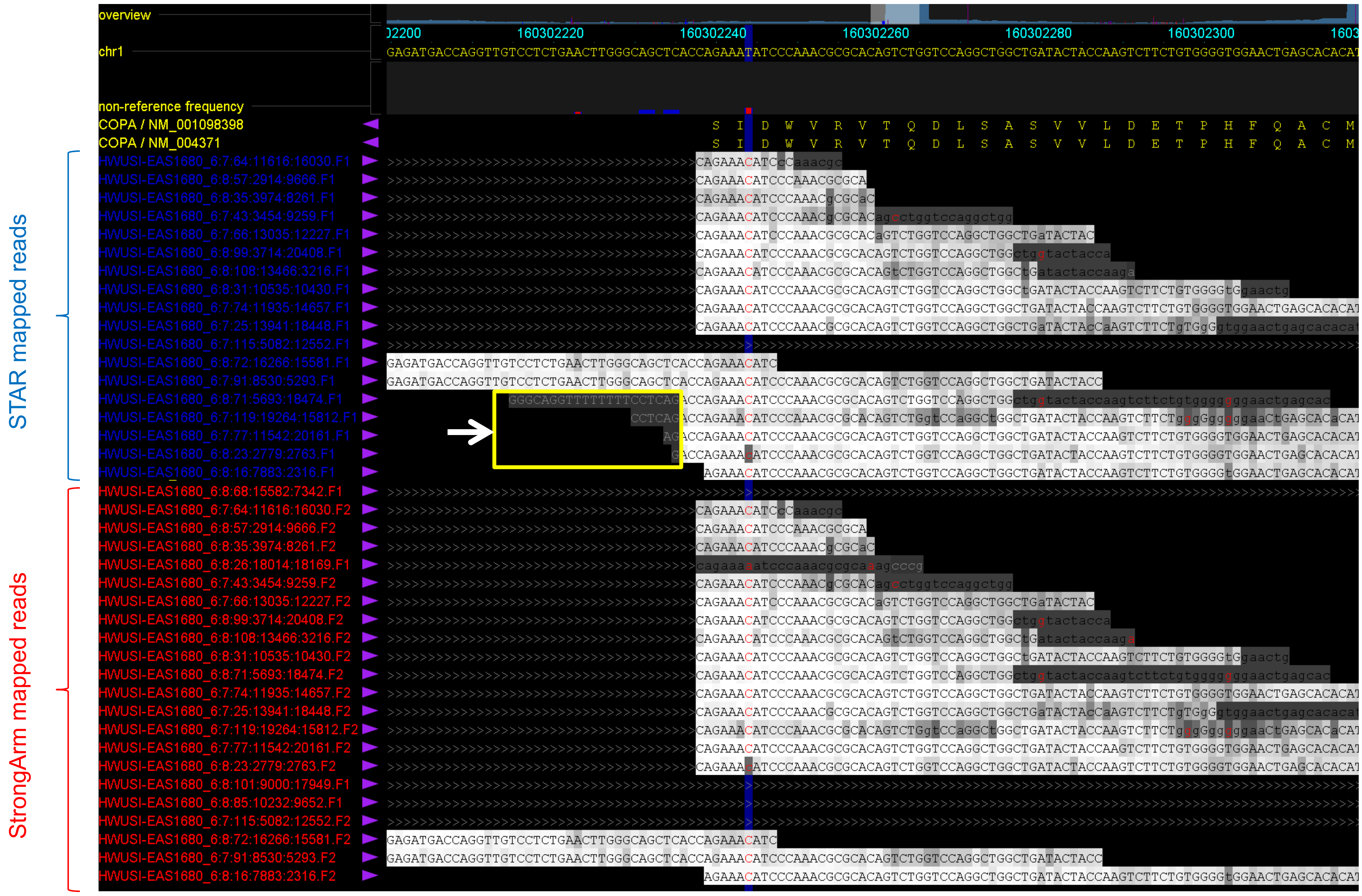

Sample SJAMLM7014_D
chr1:160302244 T>C editing
COPA gene

B

Reads with editing at the end failed mapping by STAR but mapped by StrongArm
(only reads containing edited allele are shown)

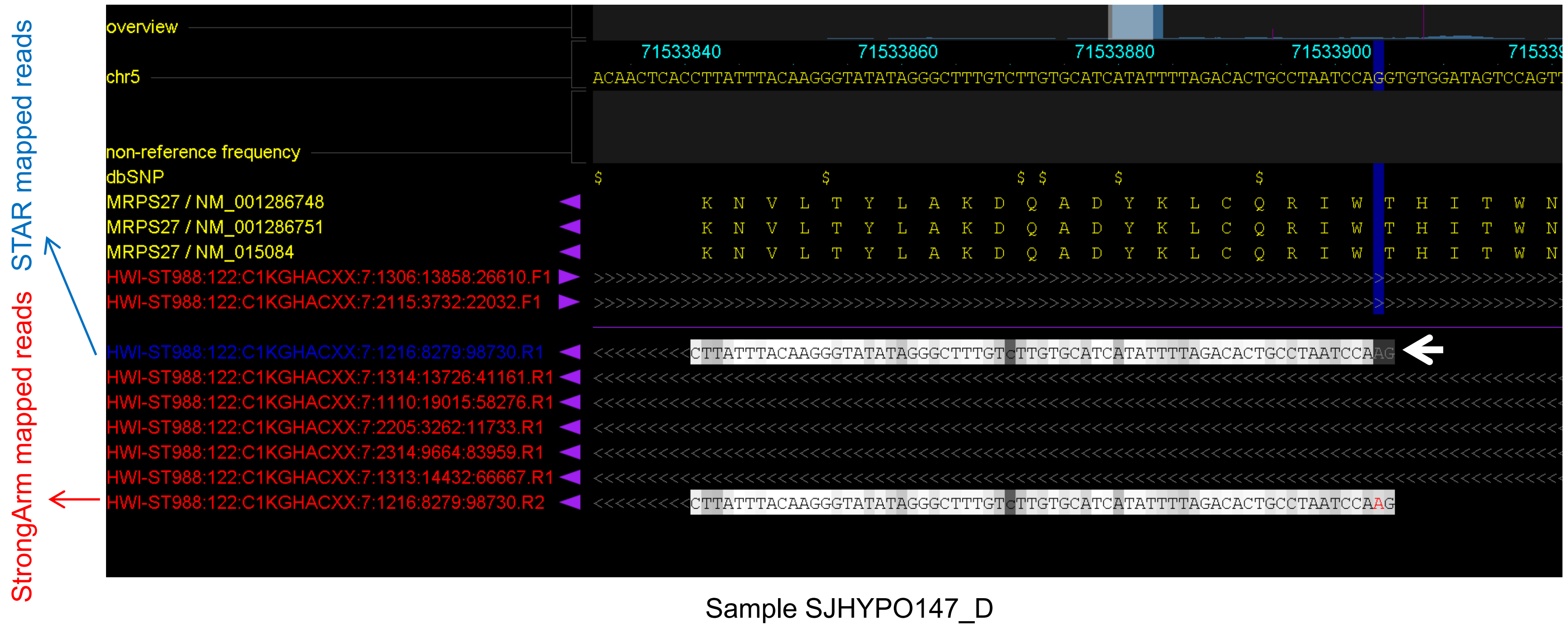

Sample SJHYPO147_D
chr5:71533904 G>A editing
MRPS27 gene

Supplementary Figure 3

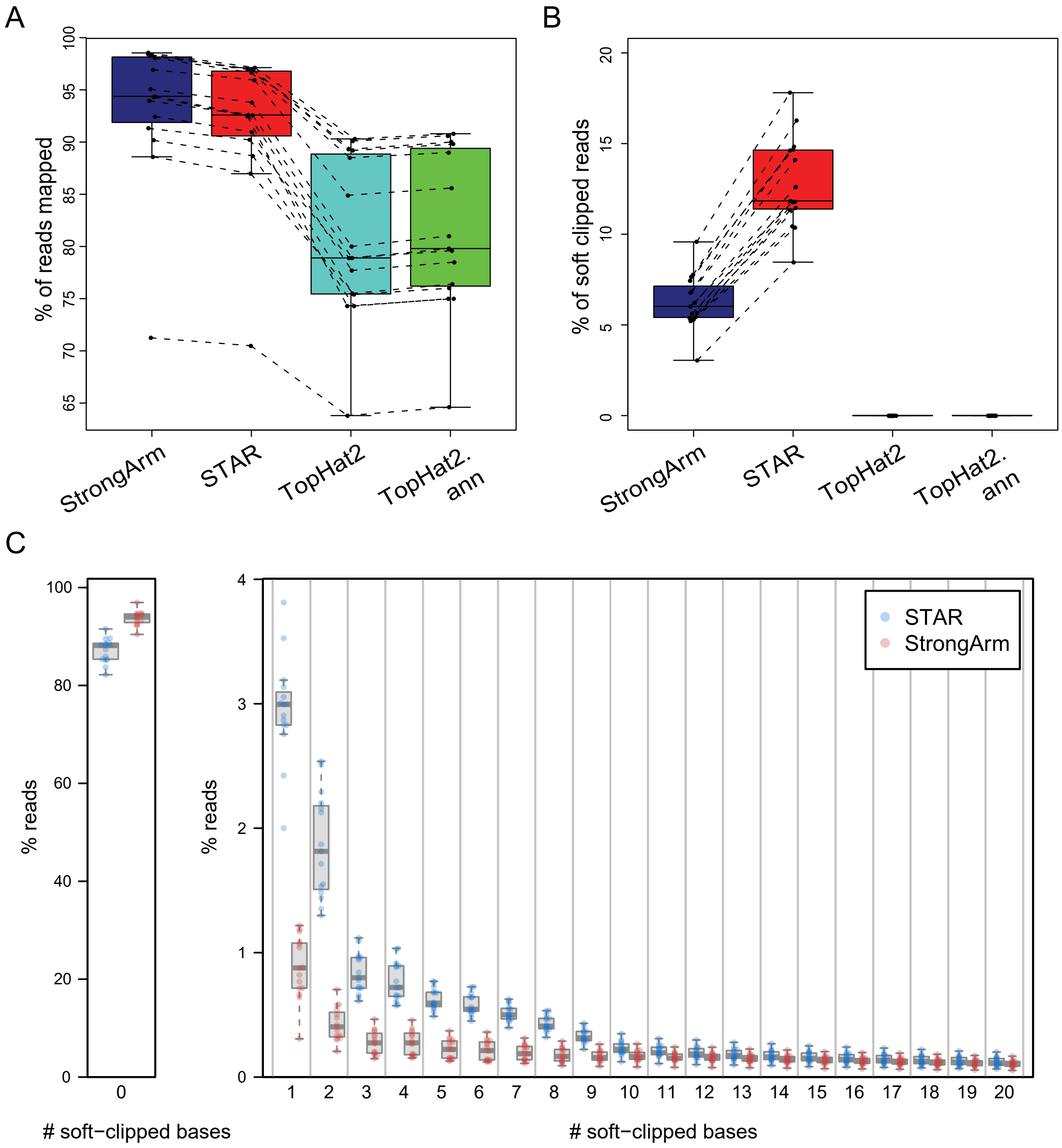

Supplementary Figure 4

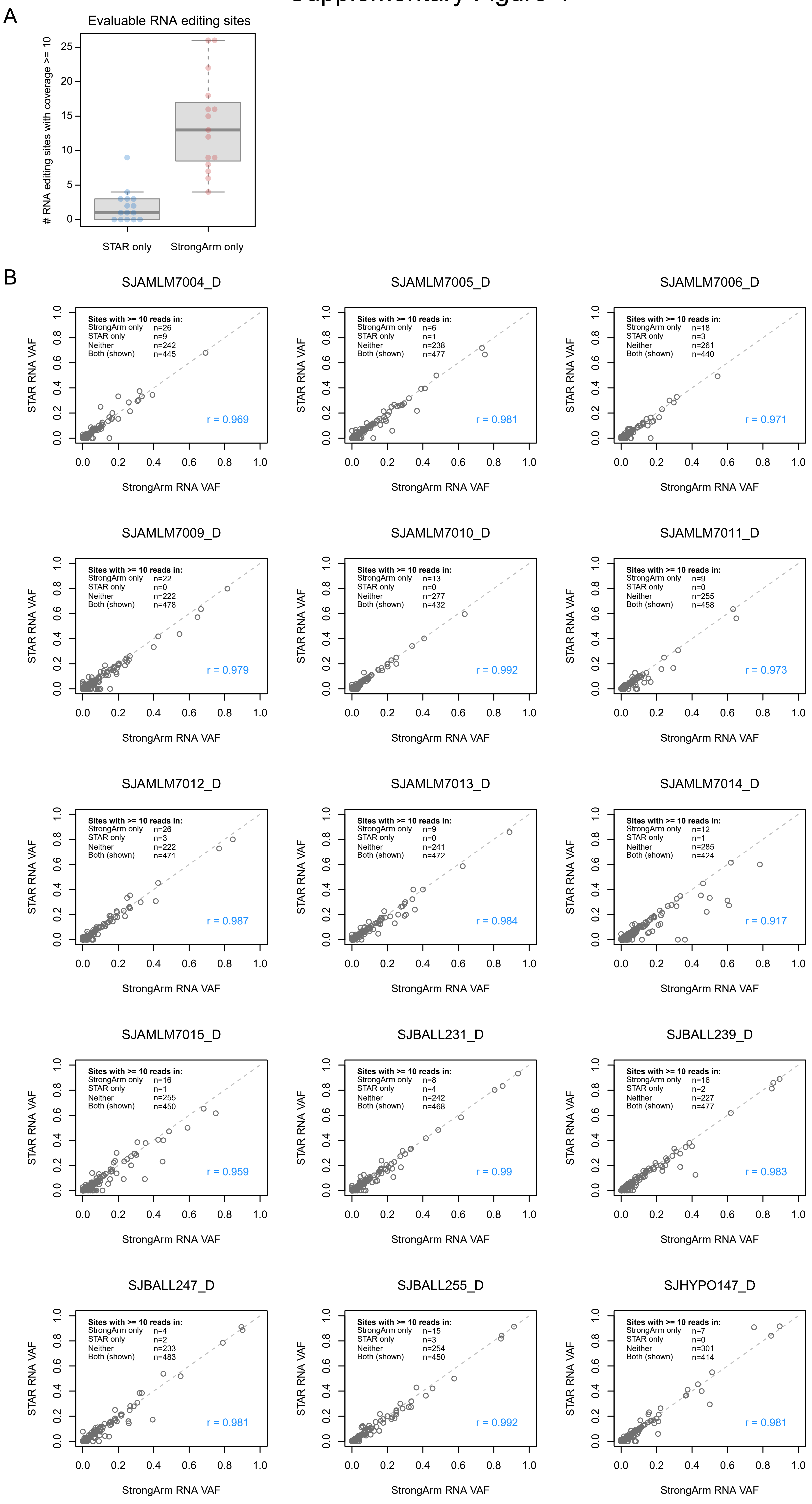

Supplementary Figure 5

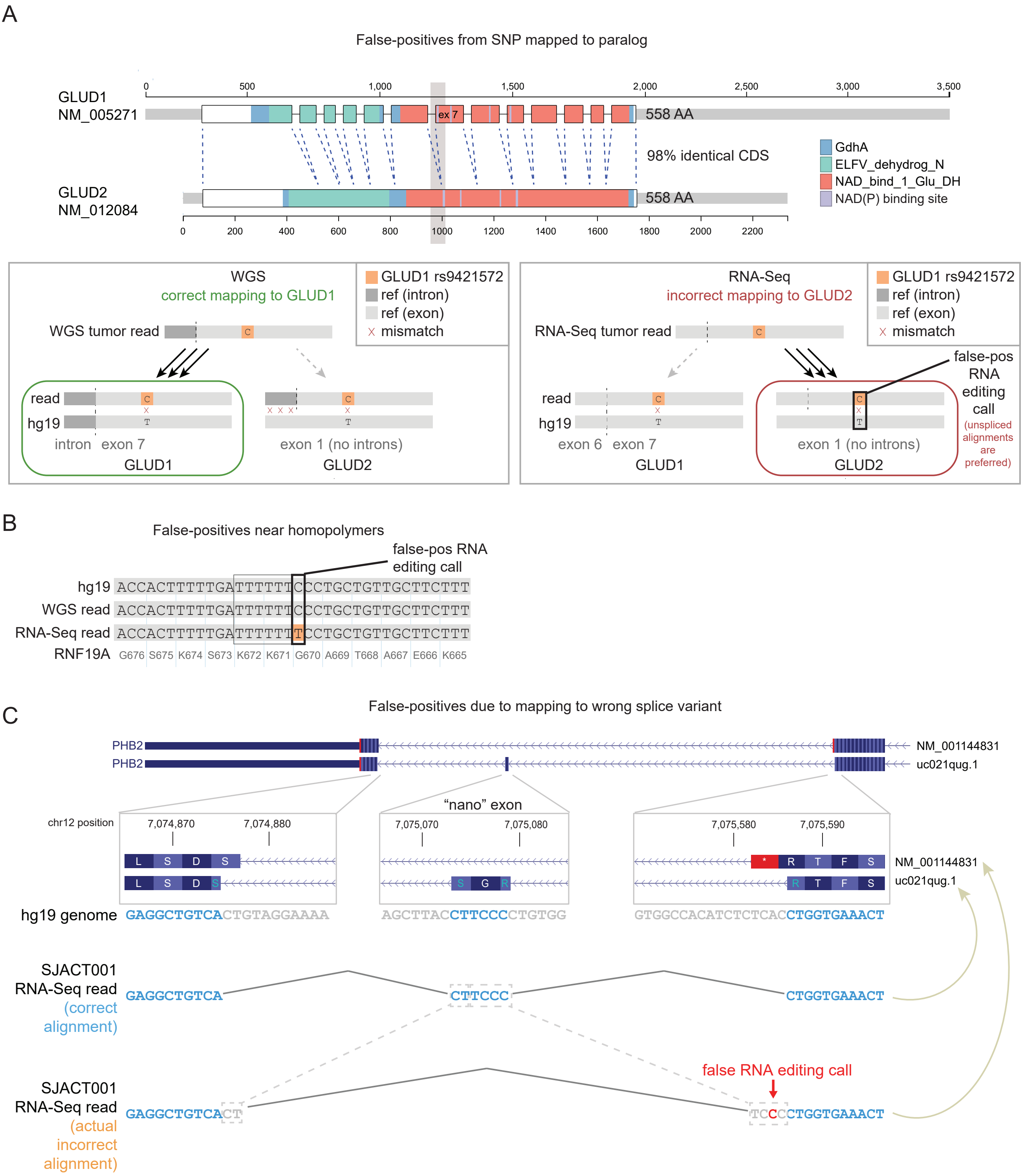

Supplementary Figure 6

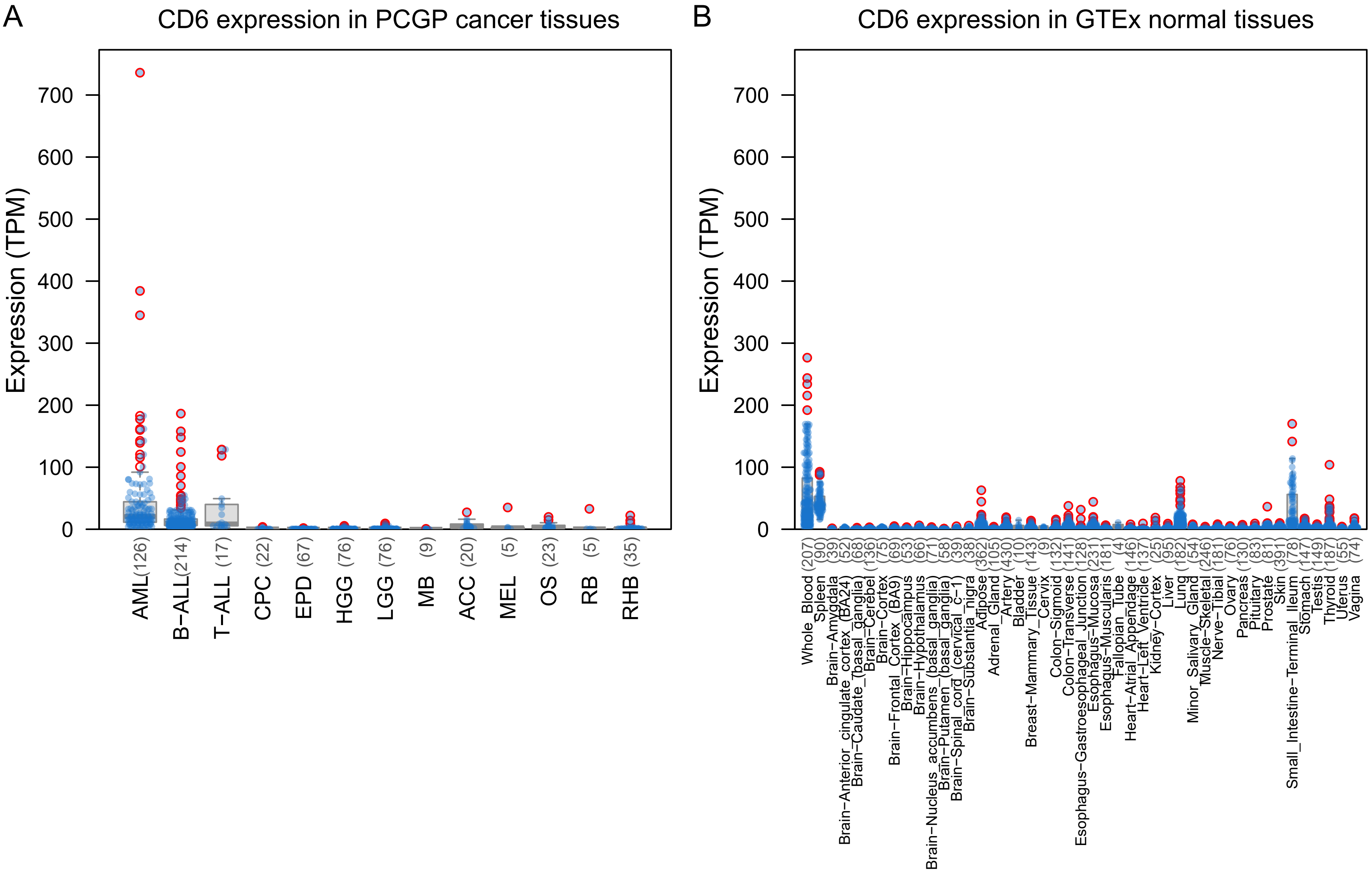

Supplement: Supplementary file 1 — Additional file 1: Supplementary Fig. 1. RNA editing analysis workflow and samples included. Workflow showing the discovery process and the criteria for sample inclusion at each step. To discover de novo potentially novel RNA editing sites (top), SNV calling was performed on samples with tumor RNA-Seq, and germline plus tumor DNA-Seq (either WGS or WES), including 717 samples (including 716 pediatric cancer samples from the PCGP and one leukemia cell line, Nalm6). After filtering and discovering RNA editing events in these 717 samples as shown in Fig. 1B, the presence of each RNA editing event discovered de novo or rescued from RADAR (n = 722 sites total) was analyzed in 954 pediatric cancer RNA-Seq samples from the PCGP, whether or not DNA-Seq was available (middle). Finally, certain samples with batch effects in RNA editing VAFs and relapsed or duplicate samples were filtered out, resulting in 711 pediatric cancer diagnosis samples with one diagnosis sample per patient (bottom). This set of 711 samples was the primary sample set shown in analyses in this study. Supplementary Fig. 2. Examples comparing alignment between StrongArm vs. STAR mapping. (A) Splice junction-adjacent reads that failed mapping by STAR (blue) but mapped by StrongArm (red) as viewed in the BAM alignment. This shows a view of one sample’s BAM files aligned by the two tools, with each row representing one read, and part of the COPA gene is shown. Soft-clipped read regions have a darker gray appearance. Gray arrows (>) indicate that part of the read is aligned elsewhere (to another exon). Non-reference sites are shown in red, including an RNA editing site (T > C). Only reads containing the edited allele are shown. (B) Read with RNA editing at the end that failed mapping (soft-clipped) by STAR but mapped by StrongArm. Coloring and other features are as in (A), except that the MRPS27 gene is shown. This shows a view of one sample’s BAM files aligned by the two tools. Only reads containing the edit [file 12885_2021_8956_MOESM1_ESM.pdf]
